# Ketogenic Diet Provided During Three Months Increases KCC2 Expression but Not NKCC1 in the Rat Dentate Gyrus

**DOI:** 10.3389/fnins.2020.00673

**Published:** 2020-07-07

**Authors:** Leticia Granados-Rojas, Karina Jerónimo-Cruz, Tarsila Elizabeth Juárez-Zepeda, Miguel Tapia-Rodríguez, Armando R. Tovar, Rodolfo Rodríguez-Jurado, Liliana Carmona-Aparicio, Noemí Cárdenas-Rodríguez, Elvia Coballase-Urrutia, Matilde Ruíz-García, Pilar Durán

**Affiliations:** ^1^Laboratorio de Neurociencias, Instituto Nacional de Pediatría, Mexico City, Mexico; ^2^Unidad de Microscopía, Instituto de Investigaciones Biomédicas, Universidad Nacional Autónoma de México, Mexico City, Mexico; ^3^Departamento de Fisiología de la Nutrición, Instituto Nacional de Ciencias Médicas y Nutrición Salvador Zubirán, Mexico City, Mexico; ^4^Departamento de Anatomía Patológica, Instituto Nacional de Pediatría, Mexico City, Mexico; ^5^Servicio de Neurología, Instituto Nacional de Pediatría, Mexico City, Mexico; ^6^Laboratorio de Biología Animal Experimental, Facultad de Ciencias, Universidad Nacional Autónoma de México, Mexico City, Mexico

**Keywords:** ketogenic diet, NKCC1, KCC2, dentate gyrus, optical fractionator, optical density, rat

## Abstract

Ketogenic diet, a high fat and low carbohydrate diet, has been used as a non-pharmacological treatment in refractory epilepsy since 1920. In recent years, it has demonstrated to be effective in the treatment of numerous neurological and non-neurological diseases. Some neurological and neuropsychiatric disorders are known to be caused by gamma-aminobutyric acid (GABA)-mediated neurotransmission dysfunction. The strength and polarity of GABA-mediated neurotransmission are determined by the intracellular chloride concentration, which in turn is regulated by cation-chloride cotransporters NKCC1 and KCC2. Currently, it is unknown if the effect of ketogenic diet is due to the modulation of these cotransporters. Thus, we analyzed the effect of a ketogenic diet on the cation-chloride cotransporters expression in the dentate gyrus. We estimated the total number of NKCC1 immunoreactive (NKCC1-IR) neuronal and glial cells by stereology and determined KCC2 labeling intensity by densitometry in the molecular and granule layers as well as in the hilus of dentate gyrus of rats fed with normal or ketogenic diet for 3 months. The results indicated that ketogenic diet provided during 3 months increased KCC2 expression, but not NKCC1 in the dentate gyrus of the rat. The significant increase of KCC2 expression could explain, at least in part, the beneficial effect of ketogenic diet in the diseases where the GABAergic system is altered by increasing its inhibitory efficiency.

## Introduction

Ketogenic diet (KD) is a high-fat, low-carbohydrate and adequate-protein diet characterized by producing a state of ketosis in the organism. The term ketogenic diet was first coined at Mayo Clinic in 1921 and derives its name from the fact that it increases the circulating concentration of the ketone bodies β-hydroxybutyrate, acetoacetate and acetone (Wheless, 2008). These ketone bodies, particularly β-hydroxybutyrate, can replace glucose as fuel for cells. The synthesis of ketone bodies begins once glycogen stores have depleted in the liver. Thus, the KD is a biochemical model of fasting, where cells use ketone bodies as energy substrate (Fedorovich et al., 2018). KD was initially employed as an effective non-pharmacological treatment for refractory epilepsy with beneficial results (Henderson et al., 2006; Levy et al., 2012). However, in recent years, it has demonstrated to be effective in the treatment of numerous neurological disorders such as traumatic brain injury (Appelberg et al., 2009; Hu et al., 2009); neuropsychiatric disorders as schizophrenia (Kraft and Westman, 2009; Kraeuter et al., 2015), autism spectrum disorder (Ahn et al., 2014; Lee et al., 2018), depression (Murphy et al., 2004; Sussman et al., 2015), anxiety (Sussman et al., 2015; Ari et al., 2016) and bipolar disorder (Phelps et al., 2013); as well as mitochondrial dysfunctions (Kang et al., 2007; Kim et al., 2010), cancer (Seyfried et al., 2012; Cohen et al., 2018), aging (Balietti et al., 2010a, b) and obesity (Paoli, 2014; Zhang et al., 2018).

Numerous neurological and neuropsychiatric disorders including autism spectrum disorder (Huberfeld et al., 2007; Di Cristo et al., 2018), schizophrenia (Gonzalez-Burgos and Lewis, 2008; Hashimoto et al., 2008), stress (Kwon et al., 2018), traumatic brain injury (Bonislawski et al., 2007) and epilepsy (Ben-Ari et al., 2012) are known to be caused by the dysfunction of the gamma-aminobutyric acid (GABA)-mediated neurotransmission (Kwon et al., 2018). It is well known that the strength and polarity of GABA-mediated neurotransmission are determined by the intracellular chloride concentration, which in turn is regulated by cation-chloride cotransporters and is indeed essential for neuronal homeostasis activity in the brain. The main chloride “exporter” is the K^+^/Cl^–^ cotransporter (KCC2), which can extrude chloride from the neuron against its concentration gradient. In opposite direction, the Na^+^/K^+^/Cl^–^ cotransporter (NKCC1) is regarded as the most active chloride “importer”. Together, KCC2 and NKCC1 are the two main transporters responsible for regulating intracellular chloride concentration (Schulte et al., 2018).

Recent studies have reported alterations of NKCC1 or KCC2 cotransporters in multiple models of neurological and psychiatric diseases, including schizophrenia (Hyde et al., 2011; Merner et al., 2016), autism (Cellot and Cherubini, 2014; Merner et al., 2015), Down syndrome (Deidda et al., 2015), epilepsy (Hübner et al., 2001; Huberfeld et al., 2007), cerebral ischemia (Jaenisch et al., 2010), tuberous sclerosis complex (Ruffolo et al., 2016), traumatic brain injury (Bonislawski et al., 2007), neuropathic pain (Cramer et al., 2008) and stress (Tsukahara et al., 2015; Kwon et al., 2018). KCC2 is down-regulated while NKCC1 is up-regulated under certain pathophysiological conditions, such as epilepsy and trauma (Wang et al., 2016). These studies underpin the importance of NKCC1 and KCC2 regulation for the homeostasis of neuronal intracellular chloride concentration and appropriate function of GABA signaling.

Some studies have shown that the efficacy of KD is manifested after 1 month of treatment in animal models. In a previous study of our group (Gómez-Lira et al., 2011), we reported that KD *per se* does not alter the expression of the cotransporters NKCC1 and KCC2 in the hippocampus after 1 month of treatment, however, Wang et al. (2016) reported that KD increases the expression of KCC2 cotransporter in the cerebral cortex after a month of diet.

The mechanism by which KD acts is not clearly understood. However, it is important to note that KD has a beneficial effect in several diseases or disorders where GABAergic system failure is involved, probably by modifying the cation-chloride cotransporters NKCC1 and KCC2 as a common mechanism. However, so far, there are no studies that have analyzed the long-term effect of KD *per se* on the expression of the cation-chloride cotransporters in the dentate gyrus. Hence, the present work was focused to analyzing the long-term effect of KD on the expression of cation-chloride cotransporters, particularly in the dentate gyrus. In view of this, the total number of NKCC1 immunoreactive (NKCC1-IR) neuronal and glial cells was estimated by stereology, while KCC2 labeling intensity was determined by optical densitometry in the molecular and granule layers and in hilus of dentate gyrus of rats after 3 months of normal diet or KD administration.

## Materials and Methods

### Animals and Diets

Male Sprague-Dawley rats were bred and maintained in controlled conditions of temperature (22–24°C), light:dark cycle (12:12 h) and relative humidity (40%). This research was performed according to the guidelines of the Official Mexican Norm (NOM-062-ZOO-1999) and are part of project 085-2010, approved by the Research Board of the National Institute of Pediatrics, registered at the Office for Human Research Protection of the NIH^1^ with number IRB00008065; the project was also approved by the Institutional Committee for the Care and Use of Laboratory Animals (CICUAL).

At postnatal day 21 (P21), rats from 8 litters were weaned and randomly divided into two groups: (1) control group (ND, *n* = 8), animals fed with a normal diet (2018S, Envigo Teklad, United States) (Table 1) and (2) experimental group (KD, *n* = 8), animals fed with a ketogenic diet (TD.96355, Envigo Teklad, United States) (Table 1). Both diets were started at weaning and maintained during 3 months. The animals had *ad libitum* access to water and food. The rats were fasted for 1 day prior to the dietary treatment. Body weight, glucose and β-hydroxybutyrate blood levels (in tail blood samples) of the animals were measured at the beginning (P21) and at the end of the treatments (P112). The glucose and the β-hydroxybutyrate concentrations were determined using a FreeStyle Optium system and glucose or β-hydroxybutyrate test strips (Abbott Laboratories).

**TABLE 1:** Nutritional composition of diets.

Macronutrients (% by weight)	Normal diet 2018S Envigo Teklad	Ketogenic diet TD.96355 Envigo Teklad
Protein	18.60	15.30
Fat	6.20	67.40
Carbohydrate	44.20	0.50
**Minerals**		
Calcium	1.00	0.90
Phosphorus	0.70	0.56
Potassium	0.60	0.54
Sodium	0.20	0.15
Chloride	0.40	0.24
Magnesium	0.20	0.10
Copper (mg/kg)	15.00	8.90
Iron (mg/kg)	200.00	54.60
Zinc (mg/kg)	70.00	56.80
Manganese (mg/kg)	100.00	87.60
Iodine (mg/kg)	6.00	0.31
Selenium (mg/kg)	0.23	0.16
Energy (kcal/g)	3.10	6.70

*2018S diet contains vitamins A, D3, E, K3, B1, B2, B6, B12, niacin, pantothenic acid, biotin, folate and choline. TD.96355 diet is supplemented with vitamin mix (Teklad, 40060).*

### Tissue Processing and Sample Collection

At the end of treatment, rats were anesthetized with sodium pentobarbital (50 mg/kg, intraperitoneally) and transcardially perfused with saline followed by 4% paraformaldehyde in phosphate buffer, 0.1 M, pH 7.4 (PFA). Brains were removed, post-fixed in PFA overnight and serially cryo-protected in 10, 20, and 30% sucrose at 4°C. Afterward, 50 μm thick coronal serial sections were obtained of all rat dentate gyrus (−1.72 to −6.84 mm posterior to Bregma, Paxinos and Watson, 2007) using a cryostat (Leica, Germany). Ventral and dorsal dentate gyrus were considered, and both hemispheres were inspected. Sections were stored in a cryoprotectant solution (25% glycerol, 25% ethylene glycol, 50% phosphate buffer 0.1 M, pH 7.4) at −20°C in 24 well plates until use. To select the sections from the serial slides per animal, we use a systematic random procedure consisting of choosing one of every eight sections that resulted in eight series of 12–15 sections of all rat dentate gyrus. One of the series was immunohistochemically processed for immunodetection of NKCC1 and other for KCC2 in each rat.

### Immunohistochemical Staining

To evaluate the expression of the cation-chloride cotransporters NKCC1 and KCC2 in ND and KD rats, an immunohistochemistry protocol was carried out using a secondary biotinylated antibody according to Brandt et al. (2010). Brain tissue sections from ND and KD rats were processed in parallel free-floating at room temperature in constant motion on a shaker. Sections were initially subjected to three-time 10-min washes with PBS, between the change of each solution and at the end. After washing with PBS, sections were subjected to 1% hydrogen peroxide in PBS during 10 min. Tissues were then incubated with 20X ImmunoDNA retriever buffer (Bio SB, United States) at 65°C for 60 min. After that, they were incubated overnight with the primary rabbit polyclonal antibodies anti-NKCC1 (1:500; Merck-Millipore, Germany, Cat. # AB3560P), or anti-KCC2 (1:2000; Merck Millipore, Germany, Cat. # 07-432), diluted in 5% horse serum (Gibco) and 3% Triton X-100 (Merck, Germany) in PBS. Both antibodies recognize their respective total protein. The next day, sections were washed and incubated with a secondary biotinylated goat anti-rabbit biotinylated IgG antibody (1:500; Vector Laboratories, United States, Cat. # BA-1000) for 2 h and subsequently incubated with avidin peroxidase complex (ABC kit; Vectastain; Vector Laboratories, United States, Cat. # Pk-4000) for 1 h. To reveal peroxidase activity, we used a nickel-intensified 3,3′-diaminobenzidine (DAB; Vector Laboratories, United States, Cat. # SK-4100) solution for 2 1/2 min. Finally, the sections were mounted on poly-L-lysine-coated slides, entellan (Merck, Germany) was added and slides covered with a glass coverslip. In additional sections, the primary antibody NKCC1 or KCC2 as well as the secondary biotinylated antibody were omitted as negative controls to assess non-specific binding. The same amount of horse serum used to replace the primary or secondary antibody resulted in lack of any staining. Evaluation of sections was performed in a blind fashion, i.e., the researcher was not aware whether sections were from ND or KD rats.

### Immunofluorescence

To further investigate the cell lineage of NKCC1-IR and KCC2-IR cells, co-staining of NKCC1 or KCC2 was realized with NeuN (a neuron-specific marker), GFAP (a marker for astrocytes) and DAPI (nuclear staining). Free floating brain sections were post-fixed in 4% PFA for 10 min. After three washes (10 min each) with PBT, the sections were incubated with 2X SSC during 60 min at 70°C and then at room temperature for 30 min. Next, sections were washed three times with PBT and then simultaneously incubated with mouse anti-NeuN (1:500; Chemicon, Merck, Germany Cat. # MAB377), chicken anti-GFAP (1:500, Merck Millipore, Germany, Cat. # AB5541), rabbit anti-NKCC1 (1:100; Merck Millipore, Germany, Cat. # AB3560P) or anti-KCC2 (1:200; Merck Millipore, Germany, Cat. # 07-432), diluted in PBT added with 3% horse serum at room temperature overnight. After this, sections were washed three times with PB and incubated during 2 h at room temperature with the following secondary antibodies: AlexaFluor 488 donkey anti-mouse (1:200, Thermo Fisher Scientific Inc., Waltham, MA, United States, Cat. # A-21202), AlexaFluor 647 donkey anti-rabbit (1:200, Thermo Fisher Scientific Inc., Waltham, MA, United States, Cat. # A-31573) and biotinylated donkey anti-chicken (1:400, Merck Millipore, Germany, Cat. # AP1948) diluted in PB. After three washes, they were incubated with Texas Red avidin D (1:200, Vector Laboratories, United States, Cat. # A-2006) and then counterstained with DAPI (Merck, Germany, Cat. # 10236276001). Finally, sections were mounted onto slides and coverslipped with anti-fading medium (Dako Fluorescence Mounting Medium, Denmark). Negative controls were done on a slide with all primary omitted but incubated with secondary antibodies; no signal was detected on this. Fluorescence images were acquired with a Nikon A1R^+^ laser scanning confocal scanning head coupled to an Eclipse Ti-E inverted microscope (Nikon Corporation, Tokyo, Japan) equipped with a motorized stage (TI-S-E, Nikon). For XY imaging, samples were sequentially excited with 647 (2.1 mW), 561 (1.05 mW), 488 (1.05 mW) and 405 (2.4 mW) laser, imaged through a CFI Plan Apo VC 60X N.A. 1.2 water immersion objective (Nikon), and evaluated with galvanometric scanner, 660LP, 600/50, 525/50, 450/50 emission filters, and GaAsP/standard detectors. Pinhole value was set at 12.77 μm. All images were captured with NIS Elements C software v. 5.00 (Nikon), and processed with Fiji software (v.1.52p) (Linkert et al., 2010; Schindelin et al., 2012).

### Stereology for NKCC1

A stereological, systematic random procedure, *optical fractionator*, (West et al., 1991) was employed for counting the number of NKCC1-IR neuronal and glial cells in dentate gyrus of both ND and KD fed rats. To achieve this, we used Stereo Investigator 9 software in a semi-automatic stereological system (MBF Bioscience, VT, United States). The counting frame size was set at 70 × 45 μm, height dissector 13 μm, and guard zones were defined at 1.5 μm from the upper and lower borders of the counting frame. Grid size was set at 300 × 300 μm, except for the granular layer, for which was set at 250 × 250 μm. Cell counting was done at 60× and the coefficient of error (Gundersen, *m* = 1) was <0.1. NKCC1-IR neuronal and glial cell number was counted in the three layers of dentate gyrus: molecular, granule and hilus. NKCC1-IR neuronal or glial cells were identified according to Brandt et al. (2010), following the criteria of size and morphologic appearance. Thus, we considered NKCC1-IR glial cells as those with small-sized profiles (<8 μm) and intensely staining, and as NKCC1-IR neural cells, those with large-sized profiles (>8 μm) and staining from slightly to considerably darker than background.

### Optical Density of KCC2

The determination of KCC2 cotransporter expression in the dentate gyrus was done through digital densitometrical analysis of the image color intensities. All images were taken with identical characteristics of acquisition (objective lens, aperture condenser, light intensity, exposure time and white balance) with a MBF-CX9000 RGB CCD camera (MBF Bioscience, VT, United States) coupled to a BX-51 microscope (Olympus Corporation, Tokyo, Japan) and Stereoinvestigator software (MBF Bioscience, VT, United States). ImageJ software (v 1.52e, Rasband, 2018) was used to perform densitometric measurements and the values obtained were expressed as optical density (OD) in arbitrary units; for each image, we converted RGB to 8-bit color depth, segmented the layers of interest and measured the relative intensity of pixels in each region. The analysis was made at 20× in series of 12–15 sections of whole dentate gyrus. Optical density of KCC2 was estimated in three regions: molecular and granule layers and hilus of the dentate gyrus. Each value of OD was normalized using background subtraction.

### Statistical Analysis

Data were probed for normal probability distributions with the Levene test and for equality of variances with Shapiro Wilk test (SPSS software, v.25), if they come from one, Student’s *t*-test was performed, otherwise, the non-parametric Mann-Whitney *U*-test was applied. Differences were considered significant at *p* < 0.05. Data were expressed as mean ± standard deviation (SD) if its statistical distribution was normal or as median with interquartile range (IQR) if they were non-parametric data.

## Results

### Body Weight

The KD was well tolerated during the 3 months of study. The body weights of both ND and KD groups at the beginning of the treatment were not statistically different, showing equality of initial conditions. Body weights of ND and KD rats continuously increased along the treatment. After 3 months of treatment there were no significant differences in body weight between the ND and KD group despite observing a slight reduction of this parameter in the KD group. The values of the mean ± SD of body weight for groups ND and KD were: 47.23 ± 2.09 and 50.25 ± 8.33 g respectively at the beginning and 478.33 ± 51.49 and 467.45 ± 63.59 g respectively at the end.

### Glucose

**FIGURE 1:**
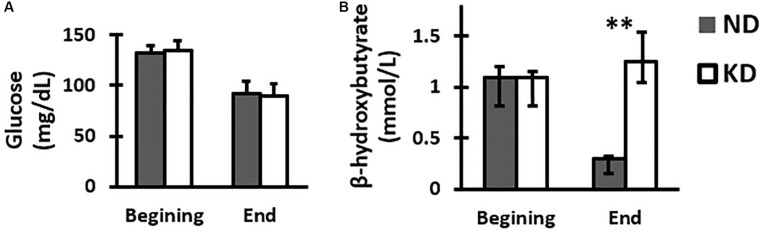
Graph of the mean and SD of glucose **(A)** and median with IQR of β-hydroxybutyrate **(B)** concentrations in peripheral blood of group feed with normal diet (ND) or ketogenic diet (KD) at the beginning and end of treatment, *n* = 8 in each group. There were no significant differences between the two groups at beginning and end in the glucose levels. However, there was a significant increase in the β-hydroxybutyrate KD group when compared with ND group at the end of treatment (^∗^*^∗^p* < 0.01, Mann-Whitney *U*-test).

Assessment of blood glucose was performed at the beginning and end of the study. The results (mean ± SD) showed that peripheral blood glucose for the groups ND and KD at the time of weaning were: 132.25 ± 10.25 and 134.37 ± 11.46 (mg/dL) respectively, and at the end of the experiment, they were 92.12 ± 6.57 and 89.75 ± 11.98 (mg/dL) respectively. There were not statistically significant differences between groups at the beginning or at the end of the experiment (Figure 1A).

### β-Hydroxybutyrate

In order to assess the effect of the ketogenic diet, ketone bodies were measured, particularly β-hydroxybutyrate. The median with IQR values of the β-hydroxybutyrate concentration in peripheral blood for the groups ND and KD at the time of weaning were 1.10 [IQR 0.9–1.3] and 1.10 [IQR 0.9–1.2] (mmol/L) respectively, and at the end of the experiment were 0.3 [IQR 0.2–0.3] and 1.25 [1.1–1.6] (mmol/L) respectively. At the beginning of the treatment, the values of this ketone body in both ND and KD groups were practically the same, showing thus equal initial conditions. However, it could be seen that at the end of the treatment, β-hydroxybutyrate concentration value for the KD group was higher than the value for ND group by 316%, which was statistically significant (*p* < 0.01, Mann-Whitney *U*-test) (Figure 1B).

### NKCC1 Immunoreactivity

**FIGURE 2:**
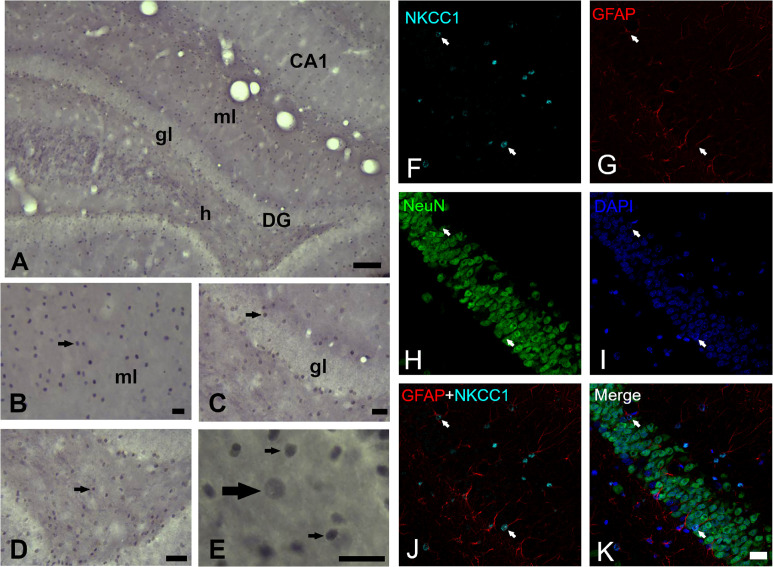
NKCC1 expression in dentate gyrus of rats fed with a normal diet. **(A)** NKCC1-IR cells in the molecular (ml) and granule (gl) layer and hilus (h) of dentate gyrus (−3.3 mm posterior to Bregma). **(B–E)** NKCC1-IR cells (arrows) were found distributed through all dentate gyrus. **(E)** Higher magnification of hilus, NKCC1 immunostaining showed predominantly small, abundant and intensely stained cells, presumptive glial cells (thin arrows) and some scarce, large and weakly stained cells, presumptive neuronal cells (thick arrow). Quadruple immunofluorescence labeling of NKCC1 with NeuN (a neuron-specific marker), GFAP (a marker for astrocytes) and DAPI (a marker for nuclei) **(F–K)**. NKCC1-IR cells were mainly astrocyte-type glial cells, abundant, small and intensely stained cells (arrows indicate on all panels, NKCC1 and GFAP immunopositive astrocytes, except in NeuN panel where arrows indicate their absence). Scale bar: 100 μm **(A)**, 25 μm **(B–D)**, 10 μm **(E)** and 20 μm **(F–K)**.

**FIGURE 3:**
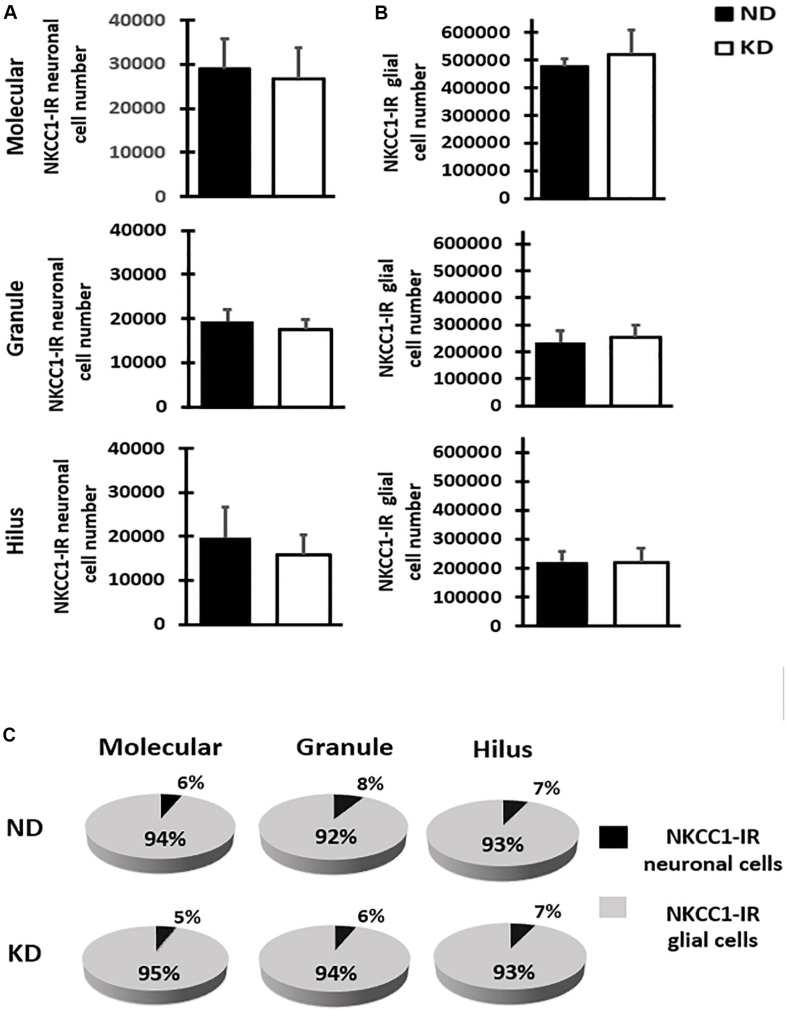
Bar graphs show mean and SD of NKCC1-IR neuronal **(A)** and glial **(B)** cells number estimated by stereology in the three layers of dentate gyrus of rats fed with normal diet (ND) or ketogenic diet (KD), *n* = 8 in each group. The estimate of cell number showed that KD does not significantly change the NKCC1-IR neuronal or glial cell number when compared with ND rats in the molecular and granule layers, and hilus of the dentate gyrus. **(C)** Fractional composition of NKCC1-IR neuronal and glial cells in the three layers of dentate gyrus in control group (ND) and ketogenic group (KD). The data are presented as a percentage of total of NKCC1-IR neuronal and glial cells in each layer.

The cytoarchitecture of the dentate gyrus did not show variations, the lamination own of dentate gyrus was preserved properly in both hemispheres. The analysis of NKCC1 staining pattern was carried out in 12–15 sections of dentate gyrus of each rat and revealed the presence of clearly stained neuronal (scarce) and glial (abundant) cells as well as processes in all layers of dentate gyrus (Figures 2A–E). It was also easy to identify and demarcate the regions of analysis: molecular layer (Figures 2A,B), granule layer (Figures 2A,C) and hilus (Figures 2A,D,E) of the dentate gyrus. NKCC1-IR neuronal or glial cells were identified following criteria of size and morphologic appearance previously described (Brandt et al., 2010). Smaller cells with intense staining were considered glial cells, and those with large size and staining from slightly to considerably darker than background were considered as neuronal cells (Figure 2E). In the fluorescent co-staining of NKCC1 with GFAP, NeuN and DAPI (Figures 2F–K), it was observed that NKCC1-IR cells were predominantly astrocyte-type glial cells (abundant, small and intensely stained cells) (Figures 2F,G,J,K). A trend of NKCC1-IR neurons reduction was observed in the KD group, however, there were no significant differences in the molecular and granule layers and hilus of dentate gyrus after 3-months of diet (Figure 3A). A trend to increase NKCC1-IR glial cells was observed in the KD group, however, there were also no significant differences in the number of NKCC1-IR glial cells between ND and KD groups (Figure 3B). A fractional composition of NKCC1-IR neuronal and glial cells in the three layers of dentate gyrus of ND and KD groups is shown in Figure 3C. In the ND group, the percentage of NKCC1-IR neuronal and glial cells was 6 and 94% respectively in the molecular layer, 8 and 92% in the granular layer, and 7 and 93% for hilus (Figure 3C). In the KD group, the percentage of NKCC1-IR neuronal and glial cells was 5 and 95% respectively for the molecular layer, 6 and 94% for granular layer and 7 and 93% for hilus (Figure 3C).

### KCC2 Immunoreactivity

**FIGURE 4:**
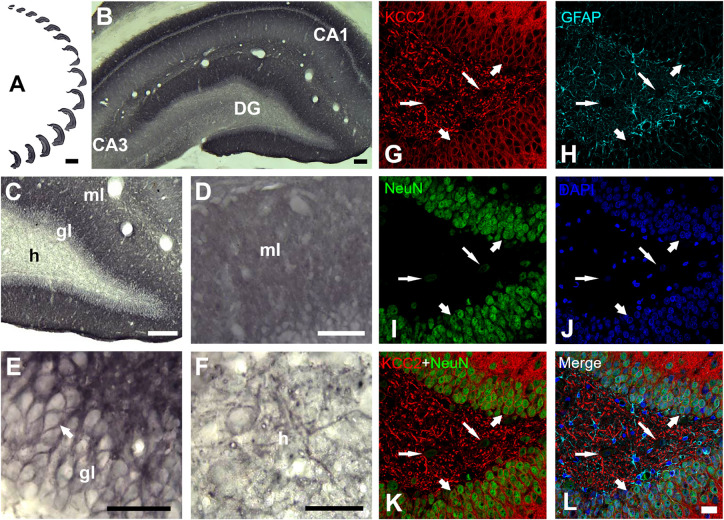
KCC2 expression in dentate gyrus of a rat fed with normal diet. **(A)** Serial sections of the whole dentate gyrus of the right hemisphere. **(B)** Panoramic and **(C)**, higher magnification view of dentate gyrus, ml, molecular layer; gl, granule layer and h, hilus. Higher magnification of molecular layer **(D)**, granule layer **(E)** and hilus **(F)**. KCC2 immunoreactivity in the granule layer was observed in the plasmalemmal region of the granule cell body (perisomal) **(E)**. In the hilus **(F)**, KCC2 expression was observed around the polymorphic cells and in neural processes. **(G–L)**, quadruple fluorescent labeling of KCC2 with NeuN (a neuron-specific marker), GFAP (a marker for astrocytes) and DAPI (a marker for nuclei) of a rat feed with normal diet. KCC2 immunoreactivity (thick arrows) was mainly observed in both cytoplasmic projections and plasmalemmal region of the granule cell body (perisomal), indicating that KCC2 is present in granular neurons **(G–L)**. KCC2-IR was also observed in neural fibers and NeuN weakly – stained cells of the hilus, probably mossy cells or hilar interneurons (thin arrows). Scale bar: 1,000 μm **(A)**, 100 μm **(B)**, 50 μm **(C)**, 25 μm **(D–F)**, 20 μm **(G–L)**.

**FIGURE 5:**
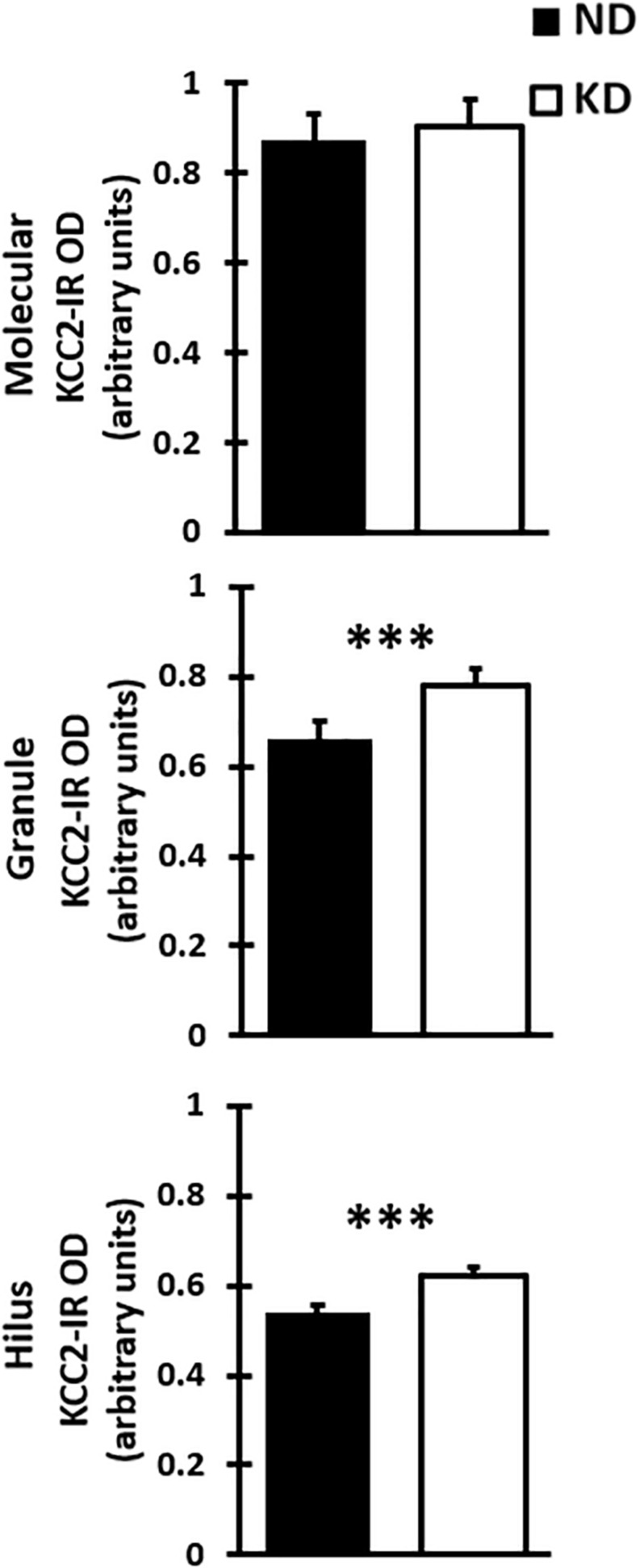
Bar graph shows mean and SD of optical density (OD) of KCC2 expression in the three layer of the dentate gyrus of rats fed with normal diet (ND) or ketogenic diet (KD), *n* = 8 in each group. The KD does not significantly change the OD when compared with ND rats in the molecular layer. However, there was a significant increase in optical density of KD group when compared with DN group in the granule layer and hilus of dentate gyrus (^∗∗^*^∗^p* < 0.000, Student’s *t*-test).

Evaluation of the cation-chloride cotransporter KCC2 expression by optical density was carried out in 12–15 sections of dentate gyrus of each rat (Figure 4A). The cytoarchitecture of the dentate gyrus and hippocampus remained unchanged between the ND and KD groups, dentate gyrus lamination was similar in both conditions (Figures 4B,C). With respect to the staining pattern, it was observed that the three layers of the dentate gyrus showed a diffuse immunoreaction, as in the rest of the hippocampus, allowing the identification and delimitation of each of the regions of analysis: molecular layer, granule layer and hilus of dentate gyrus. When the staining pattern was analyzed at a higher magnification, it was observed mainly in the molecular layer (Figure 4D). In the granule layer, the immunoreactivity of KCC2 cotransporter was around the neuronal somas i.e., in the plasma membrane, whereas the somas of the granule layer were not dyed (Figure 4E). In the hilus, KCC2 staining was observed around polymorphic cells and in neural processes (Figure 4F). The staining coloration oscillated between the black color and different shades of gray. Fluorescent co-staining of KCC2 (Figures 4G–L) with NeuN, GFAP and DAPI, showed that the staining pattern for KCC2 was similar to that obtained with immunohistochemistry with biotinylated secondary antibody, and it was abundantly located in the molecular layer. In the granule layer, KCC2 expression was around of the granular cells bodies, indicating that KCC2 was found in neurons (Figures 4G,K,L), but not in glial cells. In the hilus, KCC2-IR strongly stained neural processes and faintly stained the soma of scarce cells, probably interneurons or mossy cells. The analysis of KCC2-IR OD in the three layers of dentate gyrus showed that KD did not change KCC2 expression in the molecular layer of the dentate gyrus. However, there was a significant increase in KCC2 OD relative values in the granule layer (*p* < 0.000, Student’s *t*-test) and hilus (*p* < 0.000, Student’s *t*-test) after the treatment with KD (Figure 5).

## Discussion

The present study is the first to demonstrate, that long-lasting administration (3 months) of KD induces differential effects on the expression of cation-chloride NKCC1 and KCC2 cotransporters. Ketogenic diet modified the KCC2 cotransporter expression but not NKCC1 in the dentate gyrus of rats. Specifically, an increased KCC2 cotransporter expression was observed in the granule layer and hilus of dentate gyrus, which was not observed in the molecular layer. The ketogenic diet did not modify the NKCC1-IR neuronal and glial cells number in any of the layers analyzed. Interestingly, there were more NKCC1-IR glial cells than NKCC1-IR neural cells. The low neural expression of NKCC1 is in line with previous studies in adult rats (Brandt et al., 2010). These findings led to the conclusion that KD applied for 3 months increased the expression of KCC2 cation-chloride cotransporter in rat dentate gyrus.

These results complement our previous work, where it was shown that KD *per se* does not modify the expression of the cation-chloride cotransporters NKCC1 and KCC2 when the diet is provided for only a month (Gómez-Lira et al., 2011). Hence, the results of this work and those of our previous study indicate the necessity of long-term administration of ketogenic diet (3 months) to achieve effects in the KCC2 cotransporter.

The dentate gyrus is an important region of the hippocampal formation which has been the focus of synaptic plasticity, memory and learning process (Alkadhi, 2019), and epilepsy (Henderson et al., 2006) studies among others in rodents. So, this structure is an ideal model to elucidate the differential effects produced by KD. The dentate gyrus normally functions as a filter (Cohen et al., 2003) and GABAergic synaptic inhibition in the dentate gyrus is thought to endow this hippocampal subregion with the ability to function as a low pass filter, impeding excessive or aberrant activity from propagating into the circuit making the hippocampus to be more prone to seizure (Bonislawski et al., 2007).

Abnormalities in the dentate gyrus are likely to play a major role in the pathophysiology of various neurological diseases such as epilepsy and brain injury. Also, it is noted in experimental epilepsy and brain injury that the dentate gyrus is more excitable in these situations, probably due to alterations in GABAergic inhibition (Bonislawski et al., 2007), produced in turn by changes in the cation-chloride cotransporters.

The intracellular chloride concentration determines the strength and polarity of GABA-mediated neurotransmission, thus the efficacy of GABA-mediated inhibition depends on the low intracellular chloride concentration maintenance, regulated in turn by KCC2, which is the main chloride exporter. In this work, we brought into evidence increased KCC2 cotransporter in the dentate gyrus of rats after a treatment with KD. Further studies are needed to determine whether this increase in KCC2 expression underlies a hyperpolarizing effect of GABA-mediated neurotransmission.

The beneficial effect of KD in the diseases where GABAergic system and the dentate gyrus are damaged is probably due, at least in part, to the increase of KCC2. In addition, an increase of KCC2 in dentate gyrus may contribute in improving the function of the GABAergic system and modulate neuronal excitability, benefiting both cognitive deficits and epilepsy. This hypothesis needs to be evaluated in future studies.

In contrast, when chloride extrusion is disrupted due to decreased expression of KCC2, the intracellular chloride concentration inside the neuron increases, diminishing the driving force for GABA-mediated inhibitory currents which results in significant disinhibition (Bonislawski et al., 2007), especially in the dentate gyrus, as seen in neurological and neuropsychiatric diseases like epilepsy, traumatic brain injury, schizophrenia, autism, in addition to stress (Schulte et al., 2018). Interestingly in these diseases, the beneficial effect of ketogenic diet has been demonstrated. KD could also have positive effects on other diseases such as neuropathic pain, Rett syndrome associated with autism, tuberous sclerosis and stroke in which KCC2 reduction has been seen.

In this work, we demonstrated that KD produces an over expression of KCC2 cotransporter in neuronal cells in the rat dentate gyrus. It is possible that these cells may also exhibit an upregulation of KCC2 activity that leads to a decrease in [Cl^–^]_*i*_ and consequently, an increase the magnitude of the inhibitory response to GABA. However, future studies should be conducted to explore the effect of a ketogenic diet on NKCC1 and KCC2 phosphorylation by WNK-SPAK/OSR1 pathway [WNK (kinase with no lysine (K)), SPAK (STE20-related proline-alanine-rich kinase) as well as OSRI (oxidative stress-responsive kinasa-1)], their upstream regulatory serine-threonine kinases. WNK kinases are not only effector kinases that work in conjunction with the SPAK/OSR1 kinases to regulate cation-chloride cotransporters by phosphorylation, but may also serve as intracelular Cl^–^ sensors (Moriguchi et al., 2005; Arroyo et al., 2013; Huang et al., 2019).

At the end of the experiment, β-hydroxybutyrate peripheral blood concentration increased, indicating the effectiveness of ketogenic diet used in this work. In addition, a state of ketosis endures after 3 months of treatment. A proposed mechanism for the ketogenic diet action is through the β-hydroxybutyrate.

Some studies have reported that KD reduces seizure-like activity (Bough et al., 2003; Wang et al., 2016), and normalizes various aberrant aspects of synaptic transmission (Stafstrom et al., 1999), in addition, attenuates pathological sharp waves (Simeone et al., 2014). Bough et al. (2003), showed that ketogenic calorie-restricted diet enhances GABAergic inhibition *in vivo* in rat dentate gyrus.

In contrast, KD feeding does not affect baseline excitability in the normal hippocampus, Stafstrom et al. (1999), found that KD does not modify aspects of synaptic transmission. These differences could be due to the duration of treatment with the ketogenic diet and the structure analyzed in the brain. As reported, KD *per se* presents differential effects that affect KCC2 expression in different structures such as the cerebral cortex (Wang et al., 2016) or the dentate gyrus (results of this work). The effect also depends on the treatment time. For instance, KCC2 increases in cerebral cortex in 1 month of treatment with KD, but not in the dentate gyrus, while this effect is observed in the dentate gyrus with 3 months of treatment.

Previous studies have proposed the possibility that KD effects may reach optimum status in hyperexcitable states as in epileptic condition. Clearly, more studies of KD effects on dentate gyrus regional expression of NKCC1 and KCC2 in a model of epilepsy are required. Little is known about regional alterations in dentate gyrus after applying a KD diet for 3 months.

Wang showed that KD *per se*, as in the present work, increases the expression of KCC2 without altering NKCC1. This author evaluated the expression of NKCC1 and KCC2 in an animal model of epilepsy and found that the PTZ (pentylenetetrazol) group treated with KD presented an overexpression of KCC2, and a reduction in the expression of NKCC1. These previous evidences and the results of the present work indicate that a mechanism of KD, as a non-pharmacological treatment for the control of the epilepsy, is probably by increasing KCC2 expression in the motor cortex (Wang et al., 2016), or in our case, in the dentate gyrus. However, further research is needed to confirm this last hypothesis.

In conclusion, KD provided during 3 months increases KCC2 expression but not NKCC1 in the dentate gyrus of rat. The significant increase of KCC2 expression could explain, at least in part, the beneficial effect of KD in the diseases where the GABAergic system is altered by increasing the inhibitory efficiency thus, causing the abolition of dysfunction of the dentate gyrus.

## Data Availability Statement

The data sets generated for this study are available on request to the corresponding author.

## Ethics Statement

The animal study was reviewed and approved by Institutional Committee of Care and Use of Laboratory Animals (CICUAL) of National Institute of Pediatrics (México City).

## Author Contributions

LG-R conceived and designed the study. LG-R, KJ-C, TJ-Z, MT-R, RR-J, AT, EC-U, and PD conducted research, experiments, and data collection. LG-R, AT, NC-R, MR-G, and LC-A participated in drafting the manuscript. All authors critically revised the manuscript and gave final approval for the submitted version.

## Conflict of Interest

The authors declare that the research was conducted in the absence of any commercial or financial relationships that could be construed as a potential conflict of interest.

## Footnotes

1 http://ohrp.cit.nih.gov/search/search.aspx
